# Bis(*N*′-{(*E*)-[(2*E*)-1,3-di­phenyl­prop-2-en-1-yl­idene]amino}-*N*-ethyl­carbamimido­thio­ato-κ^2^ *N*′,*S*)zinc(II): crystal structure and Hirshfeld surface analysis

**DOI:** 10.1107/S2056989017008064

**Published:** 2017-06-13

**Authors:** Ming Yueh Tan, Karen A. Crouse, Thahira B. S. A. Ravoof, Mukesh M. Jotani, Edward R. T. Tiekink

**Affiliations:** a Department of Chemistry, Faculty of Science, Universiti Putra Malaysia, 43400 UPM Serdang, Selangor Darul Ehsan, Malaysia; b Department of Physical Sciences, Faculty of Applied Sciences and Computing, Tunku Abdul Rahman, University College, 50932 Setapak, Kuala Lumpur, Malaysia; c Department of Chemistry, Faculty of Science, Universiti Putra Malaysia, 43400 UPM Serdang, Selangor Darul Ehsan, Malaysia, Department of Chemistry, St Francis Xavier University, PO Box 5000, Antigonish, NS, Canada, B2G 2W5; d Department of Physics, Bhavan’s Sheth R. A. College of Science, Ahmedabad, Gujarat 380 001, India; e Research Centre for Crystalline Materials, School of Science and Technology, Sunway University, 47500 Bandar Sunway, Selangor Darul Ehsan, Malaysia

**Keywords:** crystal structure, zinc, hydrogen bonding, thio­semicarbazone, Hirshfeld surface analysis

## Abstract

The title Zn^II^ complex, [Zn(C_18_H_18_N_3_S)_2_], (I), features two independent but chemically equivalent mol­ecules in the asymmetric unit. In each, the thio­semicarbazonate monoanion coordinates the Zn^II^ atom *via* the thiol­ate-S and imine-N atoms, with the resulting N_2_S_2_ donor set defining a distorted tetra­hedral geometry. The five-membered ZnSCN_2_ chelate rings adopt distinct conformations in each independent mol­ecule, *i.e.* one ring is almost planar while the other is twisted about the Zn—S bond. In the crystal, the two mol­ecules comprising the asymmetric unit are linked by amine-N—H⋯N(imine) and amine-N—H⋯S(thiol­ate) hydrogen bonds *via* an eight-membered heterosynthon, {⋯HNCN⋯HNCS}. The dimeric aggregates are further consolidated by benzene-C—H⋯S(thiol­ate) inter­actions and are linked into a zigzag supra­molecular chain along the *c* axis *via* amine-N—H⋯S(thiol­ate) hydrogen bonds. The chains are connected into a three-dimensional architecture *via* phenyl-C—H⋯π(phen­yl) and π–π inter­actions, the latter occurring between chelate and phenyl rings [inter-centroid separation = 3.6873 (11) Å]. The analysis of the Hirshfeld surfaces calculated for (I) emphasizes the different inter­actions formed by the independent mol­ecules in the crystal and the impact of the π–π inter­actions between chelate and phenyl rings.

## Abstract 2

The title thio­semicarbazonate complex has the ligands coordinating the Zn^II^ centre *via* the thiol­ate S and imine N atoms in each of the two independent mol­ecules comprising the asymmetric unit, leading to N_2_S_2_ donor sets and distorted tetra­hedal geometries. The crystal features zigzag chains of mol­ecules sustained by N—H⋯N and amine-N—H⋯S hydrogen bonds.

## Chemical context   

Thio­semicarbazone mol­ecules, derived from thio­semi­carbazide, H_2_N—NH—C(=S)—NH_2_, constitute an important class of mixed hard–soft, nitro­gen–sulfur donor ligands which have been extensively investigated in their coordination chemistry towards both transition metals (Lobana *et al.*, 2009 ▸) and main group elements (Casas *et al.*, 2000 ▸). Complexes of thio­semicarbazones, including Zn^II^ complexes (Da Silva *et al.*, 2013 ▸), have been evaluated variously as potential anti-cancer (Afrasiabi *et al.*, 2003 ▸), anti-viral (Garoufis *et al.*, 2009 ▸) and anti-bacterial (Quiroga & Ranninger, 2004 ▸) therapeutics for over 50 years (Dilworth & Hueting, 2012 ▸). The inter­esting properties of their metal complexes, such as structural diversity, accessible redox activities, the ability to fine-tune ligand substitution, access to radical species, catalytic properties, distinct spectroscopic properties, *etc*. afford them many potential advantages over organic-based drugs (van Rijt & Sadler, 2009 ▸; Meggers, 2009 ▸). Recent studies have focused upon their suitability as single-source precursors for ZnS nanomaterials (Pawar *et al.*, 2017 ▸). Thiosemicarbazones can exist as thione–thiol tautomers and can bind to a metal centre in neutral or anionic forms as monodentate, bidentate or bridg­ing ligands (Viñuelas-Zahínos *et al.*, 2011 ▸). The presence of additional, suitably positioned donor atoms can increase their coordination ability/denticity, giving rise to different coordin­ation geometries, such as tetra­hedral, octa­hedral and penta­gonal-bipyramidal. (Umamatheswari *et al.*, 2011 ▸). As part of a programme investigating thio­semicarbazones and their metal complexes (Tan *et al.*, 2015 ▸), the crystal and mol­ecular structures of the title compound (I) are described, complemented by an analysis of the Hirshfeld surface.
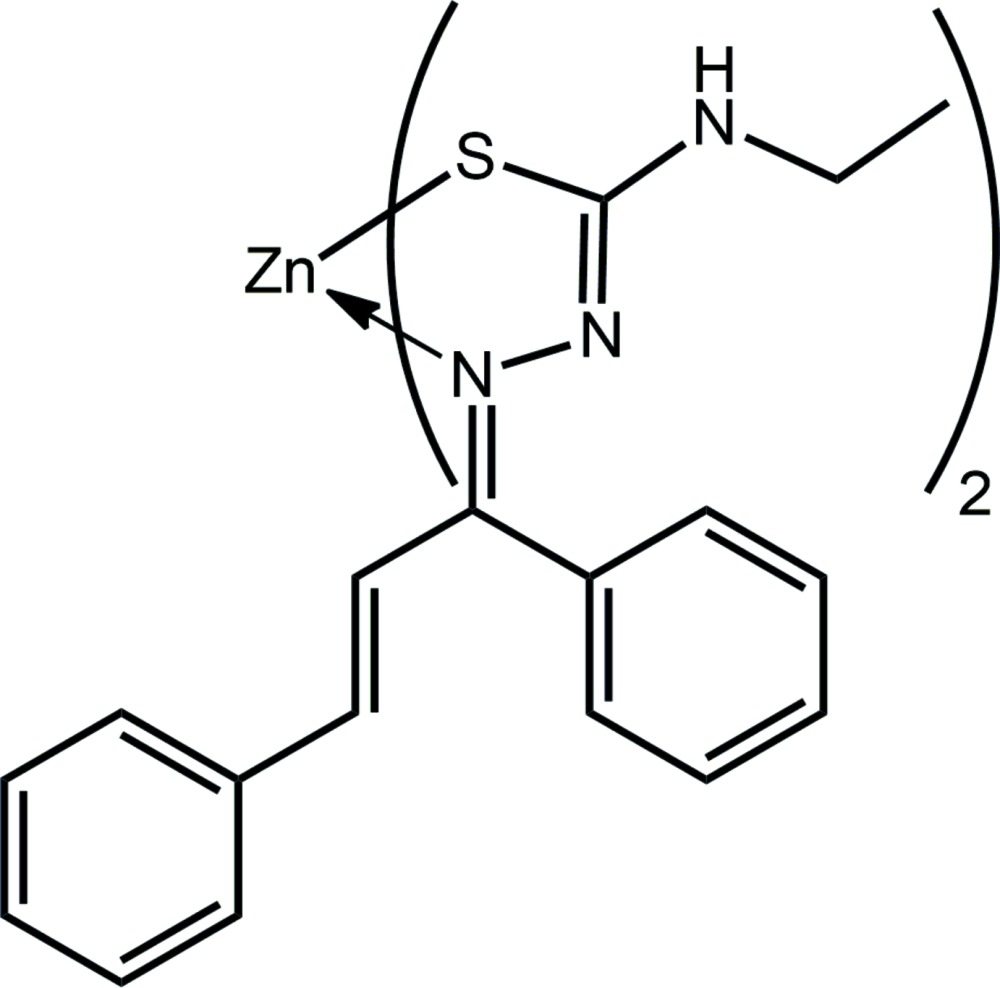


## Structural commentary   

**Figure 1:**
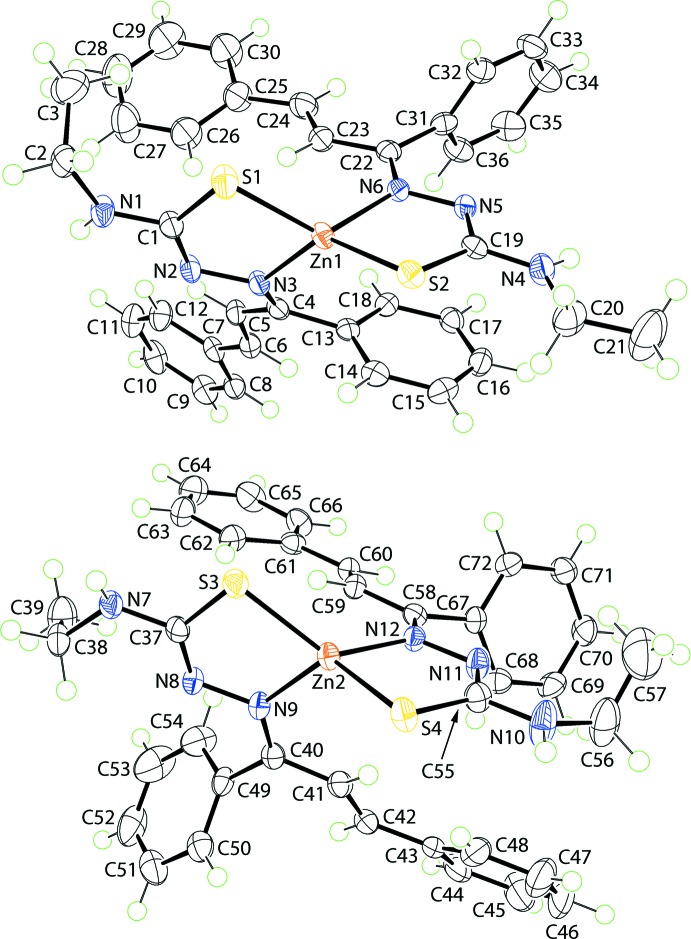
The mol­ecular structures of the two mol­ecules comprising the asymmetric unit of (I) showing the atom-labelling scheme and displacement ellipsoids at the 70% probability level.

Two independent mol­ecules comprise the asymmetric unit of (I), and these are illustrated in Fig. 1 ▸. The mono-anion derived from the thio­semicarbazone ligand is chelating, coordinating the Zn^II^ atom *via* the thiol­ate-S and imine-N atoms. Referring to Table 1 ▸, the Zn—S bond lengths in the mol­ecules span a narrow range of just over 0.01 Å, *i.e*. 2.2688 (5) Å for Zn1—S2, to 2.2827 (6) Å for Zn1—S1, whereas the Zn—N bonds show more variability, spanning a range of over 0.02 Å, *i.e*. 2.0496 (15) Å for Zn2—N12, to 2.0727 (16) Å for Zn2—N9. The similarity in bond lengths extends to the angles subtended at the Zn^II^ atoms which, for the Zn1-containing mol­ecule range from 87.00 (5)° for S2—Zn1—N6, to 134.00 (5)° for S2—Zn1—N3, *i.e*. a range of 47°; the acute angle is associated with the chelate angle. A slightly narrower range is noted for the Zn2-containing mol­ecule, *i.e*. 85.99 (5)° for S3—Zn2—N9, to 131.29 (5)° for S3—Zn2—N12, *i.e*. about 45°. The assignment of four-coordinate geometries can be qu­anti­fied by the values of τ_4_, which range from 1.00 for an ideal tetra­hedron to 0.00 for perfect square-planar geometry (Yang *et al.*, 2007 ▸). The values of τ_4_ in (I) compute to 0.70 and 0.74 for the Zn1- and Zn2-containing mol­ecules, respectively, indicating significant distortions from the ideal tetra­hedral angles. The conformation about each of the imine C=N bonds is *E*, as are the conformations about the ethyl­ene bonds, Table 1 ▸.

**Figure 2:**
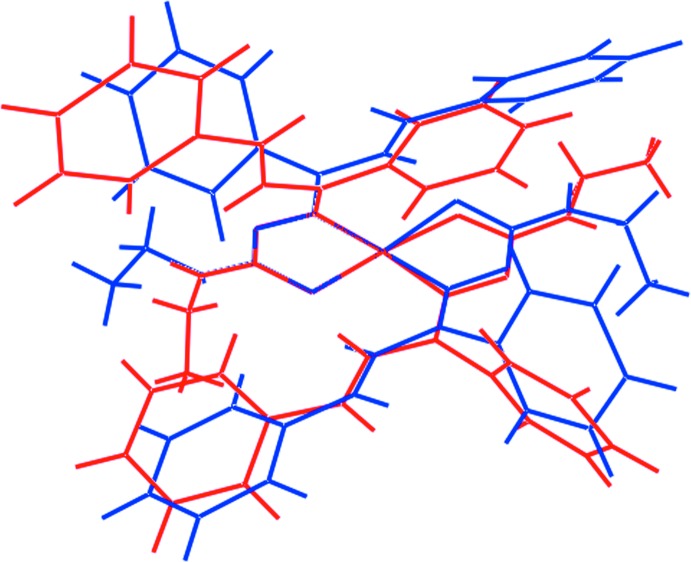
Structural overlay diagram of the two independent mol­ecules of (I): Zn1-containing mol­ecule (red image) and Zn2-containing mol­ecule (blue). The mol­ecules have been overlapped so that the two planar chelate rings are coincident.

The thio­semicarbazone ligands chelate the Zn^II^ atoms to form five-membered ZnSCN_2_ rings. The chelate rings adopt different conformations in each independent mol­ecule. For the Zn1-containing mol­ecule, the Zn1/S1/C1/N2/N3 ring is almost planar (r.m.s. deviation = 0.005 Å) but the Zn1/S2/C19/N5/N6 ring is twisted about the Zn1—S2 bond. A similar situation pertains to the Zn2-containing mol­ecule where there is a small twist about the Zn2—S3 bond in the Zn2/S3/C37/N8/N9 ring and the Zn2/S4/C55/N11/N12 ring is planar to within an r.m.s. deviation of 0.008 Å. To a first approximation, for each thio­semicarbazone ligand, all atoms but the terminal ethyl and central phenyl rings lie in a plane. This is qu­anti­fied in the dihedral angle between each five-membered chelate ring and the central and terminal rings of the prop-2-en-1-yl­idene substituent, as summarized in Table 2 ▸. The different conformations of the peripheral groups are highlighted in the overlay diagram, Fig. 2 ▸.

Some physical properties for the two independent mol­ecules in (I), calculated in *Crystal Explorer* (Wolff *et al.*, 2012 ▸) and *PLATON* (Spek, 2009 ▸), are included in Table 3 ▸. These data indicate small but significant differences between the independent mol­ecules, most notably, the Zn1-containing mol­ecule is less spherical than the Zn2-containing mol­ecule.

## Supra­molecular features   

**Figure 3:**
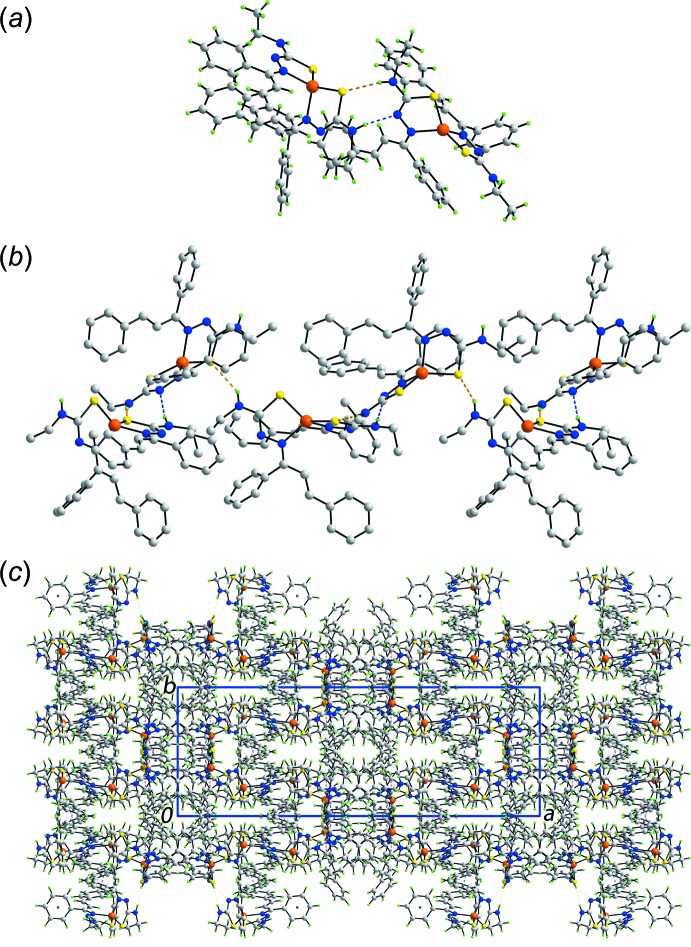
The mol­ecular packing in (I): (*a*) a view of the supra­molecular dimer sustained by amine-N—H⋯N(imine) and amine-N—H⋯S(thiol­ate) hydrogen bonds between the independent mol­ecules, shown as blue and orange dashed lines, respectively, (*b*) a view of the supra­molecular chain whereby the dimers in (*a*) are connected *via* amine-N—H⋯S(thiol­ate) hydrogen bonds and (*c*) a view of the unit-cell contents shown in projection down the *c* axis. The π–π and C—H⋯π inter­actions are shown as purple and pink dashed lines, respectively.

The most prominent feature of the mol­ecular packing is the formation of an eight-membered heterosynthon, {⋯HNCN⋯HNCS}, mediated by amine-N—H⋯N(imine) and amine-N—H⋯S(thiol­ate) hydrogen-bonds which occur between the two mol­ecules comprising the asymmetric unit, Fig. 3 ▸ *a* and Table 4 ▸. Additional benzene-C—H⋯S(thiol­ate) inter­actions stabilize the dimeric aggregate, Table 4 ▸. The dimeric aggregates thus formed are connected into a zigzag supra­molecular chain along the *c* axis *via* additional amine-N—H⋯S(thiol­ate) hydrogen-bonds, Fig. 3 ▸ *b*. Chains are connected *via* π–π inter­actions occurring between Zn2-containing mol­ecules, involving chelate rings, comprising the Zn2/S4/C55/N11/N12 atoms and phenyl (C61–C66) rings. Precedents for chelate/arene ring inter­actions have been established in the literature (Tomić *et al.*, 2006 ▸; Tiekink, 2017 ▸). In the present case, the inter-centroid separation between rings is 3.6873 (11) Å and the angle between rings is 7.89 (9)°; symmetry operation: −*x*, 1 − *y*, 1 − *z*. Additional inter­actions between chains are of the type phenyl-C—H⋯π(phen­yl) involving residues of the Zn1-containing mol­ecule exclusively, Table 4 ▸. The result of the identified inter­molecular inter­actions is the formation of a three-dimensional architecture, Fig. 3 ▸ *c*.

## Analysis of the Hirshfeld surfaces   

**Figure 4:**
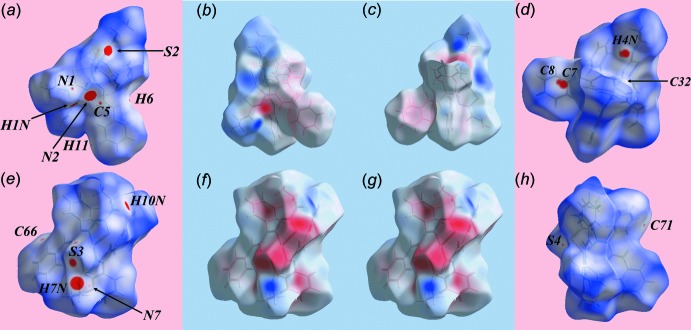
Hirshfeld surface plots for the (*a*)–(*d*) Zn1-containing mol­ecule and (*e*)–(*h*) Zn2-containing mol­ecule, plotted over *d*_norm_ in the ranges (*a*) −0.210 to +1.800 au, (*d*) −0.055 to +1.800 au, (*e*) −0.112 to +1.800 au and (*h*) −0.210 to +1.800 au and plotted over the electrostatic potential in the ranges (*b*) and (*c*) −0.118 to +0.058 au and (*f*) and (*g*) −0.046 to +0.088 au. In (*b*), (*c*), (*f*) and (*g*), the donors and acceptors are represented with blue and red regions, respectively.

**Figure 5:**
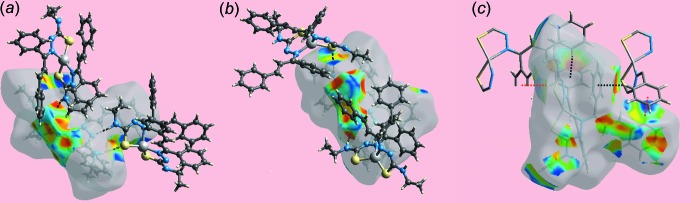
Views of Hirshfeld surface mapped over the shape-index property about a reference (*a*) Zn1-mol­ecule and (*b*) Zn2-mol­ecule, showing hydrogen bonds as black dashed lines and (*c*) Zn2-mol­ecule showing C—H⋯π and its reciprocal π⋯H—C inter­actions as red and black dotted lines, respectively.

The Hirshfeld surface calculations of (I), and for each of the Zn1- and Zn2-mol­ecules, were performed according to a recent publication on related di­thio­carbamate ligands (Jotani *et al.*, 2016 ▸). From the views of the Hirshfeld surfaces mapped over *d*_norm_ in Fig. 4 ▸ *a* and *e*, the bright-red spots near the amine-H1*N*, H7*N*, H10*N*, imime-N2 and thiol­ate-S2 and S3 atoms indicate their participation in N—H⋯N and N—H⋯S bonds between the two independent mol­ecules. In the views of the Hirshfeld surfaces mapped over electrostatic potential for the Zn1-mol­ecule in Fig. 4 ▸ *b* and *c*, and for the Zn2-mol­ecule in Fig. 4 ▸ *f* and *g*, the hydrogen-bond donors and acceptors are represented by blue and red regions, respectively. Greater insight into inter­molecular inter­actions in the crystal can be obtained by modifying the mapping range for *d*_norm_, as shown in Fig. 4 ▸ *d* and *h*, which reveals additional characteristic spots on the surface. A pair of red spots near amine-H*N*4 and near phenyl-C7 and C8 in Fig. 4 ▸ *d* indicate the presence of short inter-atomic C⋯H/H⋯C contacts in the crystal, see Table 5 ▸ for data. The tiny, faint-red spots present near the amine-N1 and N7, phenyl-C32, C66 and C77, thiol­ate-S3, ethene-C5 and H6 atoms reflect the short inter-atomic C⋯N, C⋯S and C⋯H contacts, Table 5 ▸. The comparatively weak C—H⋯S inter­action influential between the atoms of the independent mol­ecules is represented by faint-red spots near atoms H11 and S3 in Fig. 4 ▸ *a* and *e*, respectively. The immediate environments about the Zn1- and Zn2-mol­ecules within shape-index-mapped Hirshfeld surfaces highlighting hydrogen-bonding and C—H⋯π inter­actions are illustrated in Fig. 5 ▸. The N—H⋯S and N—H⋯N hydrogen bonds linking the independent mol­ecules are shown in Fig. 5 ▸ *a* and 5*b* while the C—H⋯π and their reciprocal, *i.e*. π⋯H—C, contacts involving phenyl-C8 and C32 atoms as donors and phenyl (C31–C36 and C13–C18) rings as acceptors are shown in Fig. 5 ▸ *c*.

**Figure 6:**
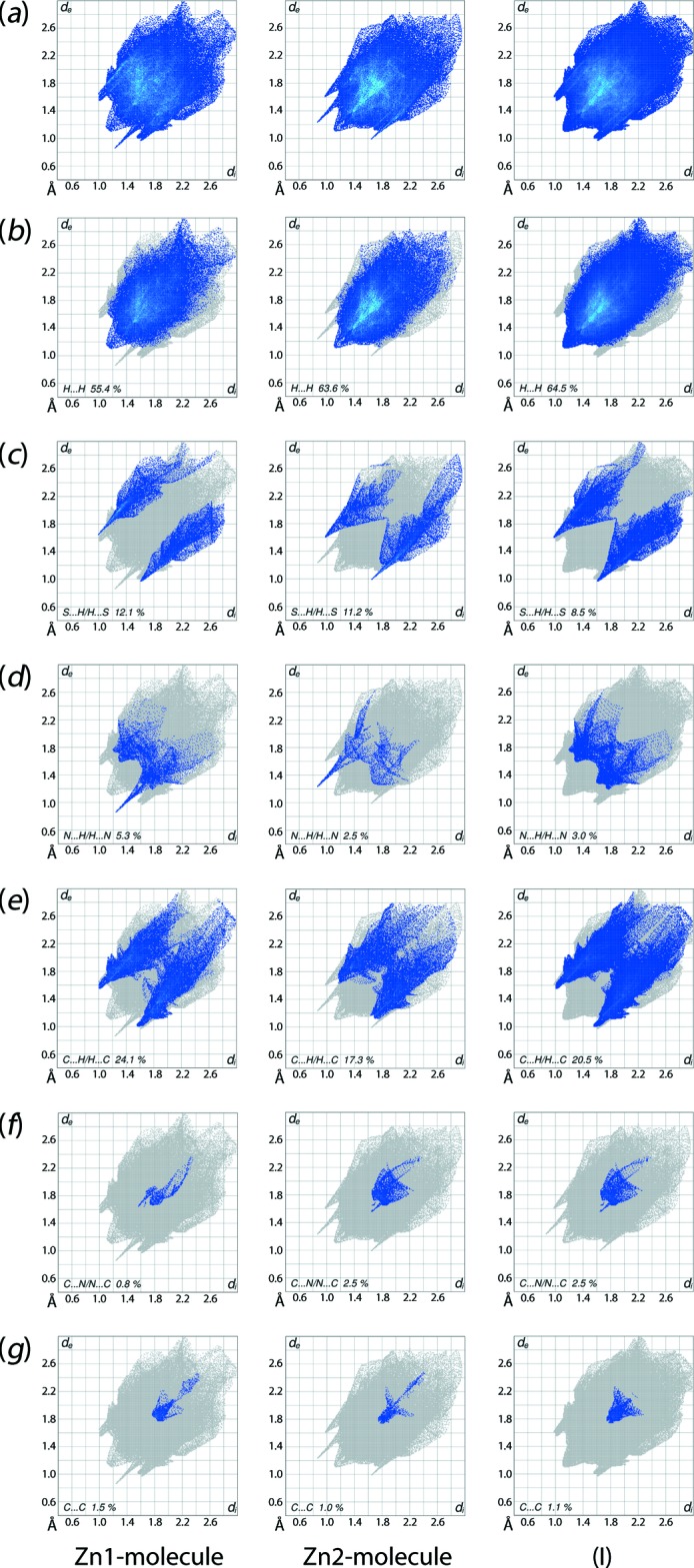
(*a*) The full two-dimensional fingerprint plots and fingerprint plots delineated into (*b*) H⋯H, (*c*) S⋯H/H⋯S, (*d*) N⋯H/H⋯H, (*e*) C⋯H/H⋯C, (*f*) C⋯N/N⋯C and (*g*) C⋯C contacts for the Zn1-and Zn2-mol­ecules and for (I).

**Figure 7:**
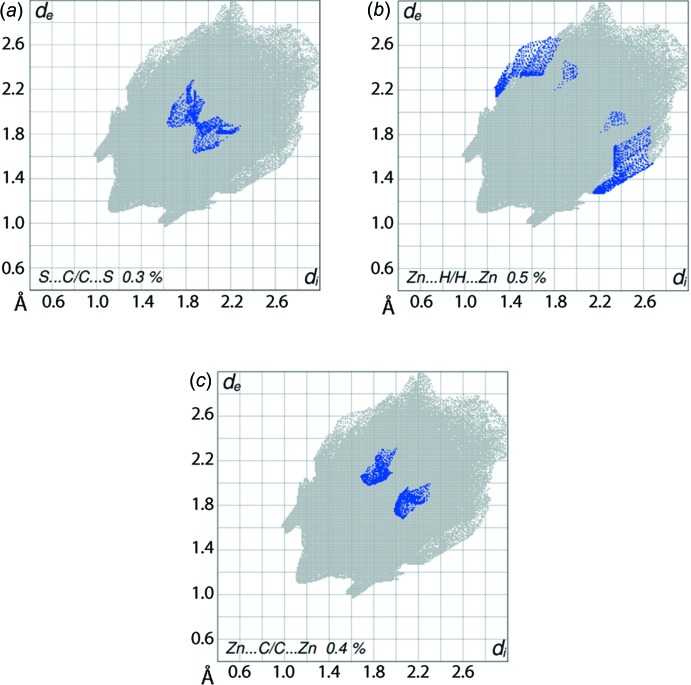
Fingerprint plots for (I) delineated into (*a*) S⋯C/C⋯2, (*b*) Zn⋯H/H⋯Zn and (*c*) Zn⋯C/C⋯Zn contacts.

The overall two-dimensional fingerprint plots for each of the Zn1- and Zn2-mol­ecules, and for the overall system, *i.e*. (I), are shown in Fig. 6 ▸ *a*. In addition, the fingerprint plots delineated into H⋯H, S⋯H/H⋯S, N⋯H/H⋯N, C⋯H/H⋯C,C⋯N/N⋯C and C⋯C contacts (McKinnon *et al.*, 2007 ▸) are illustrated in Fig. 6 ▸ *b*–*g*, respectively; their relative contributions are summarized qu­anti­tatively in Table 6 ▸. Owing to their significance upon the mol­ecular packing, the fingerprint plots delineated into C⋯S/S⋯C, Zn⋯C/C⋯Zn and Zn⋯H/H⋯Zn contacts for (I) are also illustrated in Fig. 7 ▸.

The short inter-atomic H⋯H contacts for Zn1- and Zn2-mol­ecules, Table 5 ▸, results in the peak at *d*_e_ + *d*_i_ ∼2.2 Å, appearing broader for the former and narrower for the latter mol­ecule in Fig. 6 ▸ *b*. In the fingerprint plot delineated into S⋯H/H⋯S contacts, Fig. 6 ▸ *c*, the distinct distribution of the points such as the well separated donor–acceptor regions for the Zn1-mol­ecule and the adjoining regions for the Zn2-mol­ecule are entirely consistent with the different patterns of contacts formed by these. A pair of thin spikes at *d*_e_ + *d*_i_ ∼2.7 Å in the respective fingerprint plots in the donor and acceptor regions for the Zn1- and Zn2-mol­ecules represents the N—H⋯S hydrogen bond linking the two independent mol­ecules. This pair of spikes disappears in the plot for the overall system. Another N—H⋯S hydrogen bond is recognized in the plots as differently shaped donor–acceptor regions of the Zn1- and Zn2-mol­ecules with their tips at *d*_e_ + *d*_i_ ∼2.6 Å. As the contribution from S⋯H/H⋯S contacts to the Hirshfeld surfaces of the Zn1- and Zn2-mol­ecules involves N—H⋯S hydrogen bonds and comparatively weak C—H⋯S inter­actions, the percentage contribution from these contacts to the Hirshfeld surface of the overall system is reduced to 8.5% due to disappearance of points corresponding to inter­linking N—H⋯S hydrogen bond. In Fig. 6 ▸ *d*, a pair of spikes at *d*_e_ + *d*_i_ ∼2.1 Å in the acceptor and donor regions of the Zn1- and Zn2-mol­ecules, respectively, results from the linking N—H⋯N hydrogen bond between the independent mol­ecules; the spikes disappear in the plot for the overall system.

The greater contribution, *i.e*. 24.1%, from C⋯H/H⋯C contacts to the Hirshfeld surface for the Zn1-mol­ecule *cf*. 17.3% for the Zn2-mol­ecule is due to the greater involvement of atoms of the Zn1-mol­ecule in C—H⋯π inter­actions and short inter-atomic C⋯H/H⋯C contacts, Table 5 ▸. In the fingerprint plot delineated into C⋯H/H⋯C contacts for the Zn1 mol­ecule, Fig. 6 ▸ *e*, a pair of forceps-like tips at *d*_e_ + *d*_i_ ∼2.6 Å represent a short inter-atomic C⋯H contact formed between the phenyl-C7 and amino-H7*N* atoms, Table 5 ▸. The other short inter-atomic C⋯H contacts involving the Zn1-mol­ecule are merged within the plot. Similarly, a pair of forceps-like tips in the respective plot for Zn2-mol­ecule at *d*_e_ + *d*_i_ ∼2.8 Å reflect the short inter-atomic C⋯H contact between the phenyl-C51 and -H70 atoms, with the other short contacts merged within the plot. In Fig. 6 ▸ *f*, the short inter-atomic C⋯N contacts between atoms of the Zn1- and Zn2-mol­ecules, Table 5 ▸, appear as a pair of short spikes with their tips at *d*_e_ + *d*_i_ ∼3.2 Å. The small contributions from C⋯C contacts for the Zn1- and Zn2-mol­ecules and for the overall system, Fig. 6 ▸ *g*, suggests little impact on the mol­ecular packing.

The presence of a short inter-atomic C⋯S contact between the thiol­ate-S3 and phenyl-C66 atoms is evident from the typical H-shaped plot in Fig. 7 ▸ *a* and makes a contribution of 0.6% to the Hirshfeld surface of the Zn2-mol­ecule. In the fingerprint plot delineated into Zn⋯H/H⋯Zn contacts, Fig. 7 ▸ *b*, the tips at *d*_e_ + *d*_i_ < 3.45 Å with the shape of a folded sheet with a low density of points indicate the short contacts between these atoms. The presence of π–π stacking between chelate ring Zn2/S4/C55/N11/N12 and phenyl (C61–C66) rings of Zn2-mol­ecules is evident from the presence of short inter-atomic Zn⋯C/C⋯Zn contacts, Table 5 ▸. In the fingerprint plot delineated into Zn⋯C/C⋯Zn contacts, Fig. 7 ▸ *c*, these contacts are reflected by a pair of points with an *S*-shaped distribution at around *d*_e_ + *d*_i_ ∼1.9 to 2.1 Å. This π–π stacking is also apparent from the small but effective contributions from Zn⋯C/C⋯Zn and C⋯N/N⋯C contacts to the Hirshfeld surface of the Zn2-mol­ecule, Table 6 ▸.

## Database survey   

An analysis of the Cambridge Crystallographic Database (Groom *et al.*, 2016 ▸) indicates there are nine literature precedents for the structure of (I), *i.e*. of general formula Zn[SC(NH*R*)=NN=C*R*′*R*′′]_2_ reflecting the inter­est in this class of compound. All of the structures resemble the mol­ecular geometry described above for (I). The substituents at the hydrazone-C atom can be equivalent and alkyl, *i.e. R*′ = *R*′′ = Me for the *R* = Ph compound (Tan *et al.*, 2009 ▸), or aryl, *i.e. R*′ = *R*′′ = Ph for the *R* = 3-FPh compound (Ferraz *et al.*, 2012 ▸) or mixed alk­yl/aryl, *i.e. R*′ = Me and *R*′′ = Ph for the *R* = Ph compound (Wang *et al.*, 2009 ▸); the latter structure has two mol­ecules in the asymmetric unit. The *R*′ and *R*′′ groups can be part of a ring, *e.g*. cyclo­hexyl in the structure with *R* = Me (Vikneswaran *et al.*, 2016 ▸). In most examples, the N-bound group is aryl with the exceptions being the aforementioned structure and the cyclo­pentyl analogue (Vikneswaran *et al.*, 2016 ▸). Clearly, there is immense scope for derivatization of these species which may assist in the optimization of their biological properties.

## Synthesis and crystallization   

Analytical grade reagents were used as procured without further purification. Equimolar qu­anti­ties of 4-ethyl-3-thio­semicarbazide (1.1919 g, 0.01 mol) and 1,3-di­phenyl­prop-2-en-1-one (2.0826 g, 0.01 mol) were dissolved in heated absolute ethanol (30 ml) separately and the mixtures were mixed with stirring. About five drops of concentrated hydro­chloric acid were added to the mixture to catalyse the reaction. The reaction mixture was kept under heating and stirring for about 10 mins, followed by stirring for 1 h at room temperature. The resulting yellow precipitate was filtered off, washed with chilled absolute ethanol and dried *in vacuo*. The resulting precipitate, *N*-ethyl-*N*-(1,3-diphenyl-2-propen-1-one)thio­semi­carbazide (0.3090, 0.01 mol), was used without further purification and was dissolved in heated absolute ethanol (50 ml). Zn(CH_3_COO)_2_·2H_2_O (0.1098 g, 0.50 mmol) was dissolved separately in heated absolute ethanol (30 ml) and then added into an ethano­lic *N*-ethyl-*N*-(1,3-diphenyl-2-propen-1-one)thio­semicarbazide solution. The mixture was heated and stirred for about 10 mins, followed by stirring for 1 h at room temperature. The obtained yellow precipitate was filtered, washed with cold ethanol and dried *in vacuo*. Single crystals were grown at room temperature from the slow evaporation of a solution of di­methyl­formamide and aceto­nitrile (1:1 *v*/*v* 20 ml).

## Refinement   

Crystal data, data collection and structure refinement details are summarized in Table 7 ▸. The carbon-bound H atoms were placed in calculated positions (C—H = 0.95–0.99 Å) and were included in the refinement in the riding-model approximation, with *U*_iso_(H) set to 1.2–1.5*U*_eq_(C). The nitro­gen-bound H atoms were located in a difference-Fourier map but were refined with a distance restraint of N—H = 0.88±0.01 Å, and with *U*_iso_(H) set to 1.2*U*_eq_(N).

## Supplementary Material

Crystal structure: contains datablock(s) I, global. DOI: 10.1107/S2056989017008064/hb7684sup1.cif

Structure factors: contains datablock(s) I. DOI: 10.1107/S2056989017008064/hb7684Isup2.hkl

CCDC reference: 1553218

Additional supporting information:  crystallographic information; 3D view; checkCIF report

## Figures and Tables

**Table 1:** Selected geometric parameters (Å, °)

Zn1—N6	2.0522 (16)	Zn2—N12	2.0496 (15)
Zn1—N3	2.0528 (16)	Zn2—N9	2.0727 (16)
Zn1—S2	2.2688 (5)	Zn2—S3	2.2707 (6)
Zn1—S1	2.2827 (6)	Zn2—S4	2.2823 (5)
N2—C1	1.323 (2)	N8—C37	1.311 (3)
N3—C4	1.315 (2)	N9—C40	1.307 (3)
N5—C19	1.321 (2)	N11—C55	1.309 (2)
N6—C22	1.311 (2)	N12—C58	1.308 (2)
C5—C6	1.342 (3)	C41—C42	1.339 (3)
C23—C24	1.336 (3)	C59—C60	1.344 (3)
			
S1—Zn1—S2	118.67 (2)	S3—Zn2—S4	124.97 (2)
S1—Zn1—N3	87.25 (5)	S3—Zn2—N9	85.99 (5)
S1—Zn1—N6	126.78 (5)	S3—Zn2—N12	131.29 (5)
S2—Zn1—N3	134.00 (5)	S4—Zn2—N9	124.42 (5)
S2—Zn1—N6	87.00 (5)	S4—Zn2—N12	87.23 (5)
N3—Zn1—N6	107.95 (6)	N9—Zn2—N12	105.85 (6)

**Table 2:** Selected dihedral angles (°) for (I)

Dihedral angle	Zn1,S1-ring	Zn1,S2-ring	Zn2,S3-ring	Zn2,S4-ring
Zn,*S*,*C*,*N*_2_/central phen­yl	74.54 (8)	71.88 (8)	64.79 (9)	64.53 (8)
Zn,*S*,*C*,*N*_2_/terminal phen­yl	28.13 (8)	20.17 (10)	33.66 (11)	7.89 (9)
Central phen­yl/terminal phen­yl	62.67 (10)	82.41 (11)	84.36 (13)	66.04 (10)

**Table 3:** A comparison of some physical properties of the independent mol­ecules comprising the asymmetric unit of (I)

Mol­ecule	volume, *V* (Å^3^)	area, *A* (Å^2^)	*A*:*V*	globularity, *G*	asphericity, *Ω*
Zn1-mol­ecule	847.78	646.01	0.762	0.671	0.062
Zn2-mol­ecule	853.45	615.74	0.722	0.707	0.065

**Table 4:** Hydrogen-bond geometry (Å, °) *Cg*1 and *Cg*2 are the centroids of the C31–C36 and C13—C18 rings, respectively.

*D*—H⋯*A*	*D*—H	H⋯*A*	*D*⋯*A*	*D*—H⋯*A*
N1—H1*N*⋯S3	0.87 (2)	2.65 (2)	3.5077 (19)	170 (2)
N7—H7*N*⋯N2	0.87 (2)	2.10 (2)	2.941 (2)	164 (2)
N10—H10*N*⋯S2^i^	0.87 (1)	2.59 (2)	3.318 (2)	142 (2)
C11—H11⋯S4	0.95	2.86	3.715 (2)	151
C8—H8⋯*Cg*1^ii^	0.95	2.73	3.608 (2)	154
C32—H32⋯*Cg*2^iii^	0.95	2.64	3.532 (2)	157

Symmetry codes: (i) 
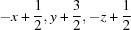
; (ii) 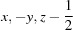; (iii) 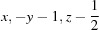.

**Table 5:** Summary of short inter-atomic contacts (Å) in (I)

Contact	distance	symmetry operation
Zn1⋯H17	3.44	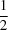 − *x*, − 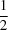 + *y*, 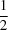 − *z*
Zn2⋯H12	3.41	*x*, *y*, *z*
Zn2⋯C11	3.942 (2)	*x*, *y*, *z*
Zn2⋯C12	3.906 (2)	*x*, *y*, *z*
Zn2⋯C65	3.735 (2)	−*x*, 1 − *y*, 1 − *z*
Zn2⋯C66	3.938 (2)	−*x*, 1 − *y*, 1 − *z*
H3*A*⋯H30	2.27	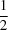 − *x*, 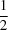 − *y*, 1 − *z*
H53⋯H53	2.23	−*x*, *y*, 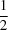 − *z*
H45⋯H54	2.37	−*x*, 2 − *y*, 1 − *z*
C5⋯N7	3.214 (2)	*x*, *y*, *z*
C71⋯N1	3.223 (3)	−*x*, 1 − *y*, 1 − *z*
C66⋯S3	3.415 (2)	*x*, 1 − *y*, 1 − *z*
C3⋯H30	2.87	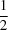 − *x*, 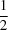 − *y*, 1 − *z*
C6⋯H4*N*	2.816 (15)	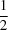 − *x*, 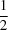 + *y*, 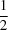 − *z*
C7⋯H4*N*	2.570 (12)	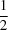 − *x*, 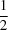 + *y*, 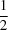 − *z*
C8⋯H4*N*	2.652 (15)	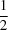 − *x*, 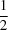 + *y*, 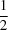 − *z*
C19⋯H18	2.85	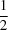 − *x*, − 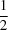 + *y*, 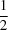 − *z*
C32⋯H6	2.74	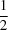 − *x*, − 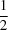 + *y*, 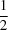 − *z*
C33⋯H6	2.87	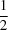 − *x*, − 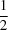 + *y*, 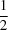 − *z*
C43⋯H3*B*	2.85	*x*, 1 + *y*, *z*
C44⋯H2*B*	2.87	*x*, 1 + *y*, *z*
C48⋯H3*B*	2.82	*x*, 1 + *y*, *z*
C51⋯H70	2.80	−*x*, 2 − *y*, 1 − *z*
C60⋯H45	2.87	−*x*, 2 − *y*, 1 − *z*

**Table 6:** Percentage contributions of inter-atomic contacts to the Hirshfeld surfaces for the Zn1-mol­ecule, Zn2-mol­ecule and (I)

Contact	distance	symmetry operation	
	Zn1-mol­ecule	Zn2-mol­ecule	(I)
H⋯H	55.4	63.6	64.5
S⋯H/H⋯S	12.1	11.2	8.5
N⋯H/H⋯N	5.3	2.5	3.0
C⋯H/H⋯C	24.1	17.3	20.5
C⋯N/N⋯C	0.8	2.5	1.2
C⋯C	1.5	1.0	1.1
C⋯S/S⋯C	0.0	0.6	0.3
Zn⋯H/H⋯Zn	0.8	0.6	0.5
Zn⋯C/C⋯Zn	0.0	0.7	0.4

**Table 7:** Experimental details

Crystal data	
Chemical formula	[Zn(C_36_H_36_N_6_S_2_)]
*M*_r_	682.20
Crystal system, space group	Monoclinic, *C*2/*c*
Temperature (K)	100
*a*, *b*, *c* (Å)	38.3604 (9), 13.6382 (3), 26.3548 (6)
β (°)	91.069 (2)
*V* (Å^3^)	13785.6 (5)
*Z*	16
Radiation type	Mo *K*α
μ (mm^−1^)	0.87
Crystal size (mm)	0.30 × 0.30 × 0.30

Data collection	
Diffractometer	Agilent Technologies SuperNova Dual diffractometer with Atlas detector
Absorption correction	Gaussian (*CrysAlis PRO*; Agilent, 2012 ▸)
*T*_min_, *T*_max_	0.782, 0.830
No. of measured, independent and observed [*I* > 2σ(*I*)] reflections	37452, 15816, 12855
*R*_int_	0.030
(sin θ/λ)_max_ (Å^−1^)	0.650

Refinement	
*R*[*F* ^2^ > 2σ(*F* ^2^)], *wR*(*F* ^2^), *S*	0.037, 0.084, 1.04
No. of reflections	15816
No. of parameters	827
No. of restraints	4
H-atom treatment	H atoms treated by a mixture of independent and constrained refinement
Δρ_max_, Δρ_min_ (e Å^−3^)	1.24, −0.48

Computer programs: *CrysAlis PRO* (Agilent, 2012 ▸), *SHELXS97* (Sheldrick, 2008 ▸), *SHELXL2014/7* (Sheldrick, 2015 ▸), *ORTEP-3 for Windows* (Farrugia, 2012 ▸), *QMol* (Gans & Shalloway, 2001 ▸) and *DIAMOND* (Brandenburg, 2006 ▸), *publCIF* (Westrip, 2010 ▸).

## supplementary crystallographic information

### Crystal data

**Table:**

[Zn(C_36_H_36_N_6_S_2_)]	*F*(000) = 5696
*M**_r_* = 682.20	*D*_x_ = 1.315 Mg m^−^^3^
Monoclinic, *C*2/*c*	Mo *K*α radiation, λ = 0.71073 Å
*a* = 38.3604 (9) Å	Cell parameters from 15819 reflections
*b* = 13.6382 (3) Å	θ = 3.1–27.5°
*c* = 26.3548 (6) Å	µ = 0.87 mm^−^^1^
β = 91.069 (2)°	*T* = 100 K
*V* = 13785.6 (5) Å^3^	Cube, yellow
*Z* = 16	0.30 × 0.30 × 0.30 mm

### Data collection

**Table:**

Agilent Technologies SuperNova Dual diffractometer with Atlas detector	15816 independent reflections
Radiation source: SuperNova (Mo) X-ray Source	12855 reflections with *I* > 2σ(*I*)
Mirror monochromator	*R*_int_ = 0.030
Detector resolution: 10.4041 pixels mm^-1^	θ_max_ = 27.5°, θ_min_ = 3.1°
ω scan	*h* = −49→48
Absorption correction: gaussian (CrysAlis PRO; Agilent, 2012)	*k* = −17→14
*T*_min_ = 0.782, *T*_max_ = 0.830	*l* = −34→22
37452 measured reflections	

### Refinement

**Table:**

Refinement on *F*^2^	4 restraints
Least-squares matrix: full	H atoms treated by a mixture of independent and constrained refinement
*R*[*F*^2^ > 2σ(*F*^2^)] = 0.037	*w* = 1/[σ^2^(*F*_o_^2^) + (0.0268*P*)^2^ + 19.4617*P*] where *P* = (*F*_o_^2^ + 2*F*_c_^2^)/3
*wR*(*F*^2^) = 0.084	(Δ/σ)_max_ = 0.002
*S* = 1.04	Δρ_max_ = 1.24 e Å^−^^3^
15816 reflections	Δρ_min_ = −0.48 e Å^−^^3^
827 parameters	

### Special details

**Table:**

Geometry. All esds (except the esd in the dihedral angle between two l.s. planes) are estimated using the full covariance matrix. The cell esds are taken into account individually in the estimation of esds in distances, angles and torsion angles; correlations between esds in cell parameters are only used when they are defined by crystal symmetry. An approximate (isotropic) treatment of cell esds is used for estimating esds involving l.s. planes.

### Fractional atomic coordinates and isotropic or equivalent isotropic displacement parameters (Å^2^)

**Table:**

	*x*	*y*	*z*	*U*_iso_*/*U*_eq_	
Zn1	0.18173 (2)	0.22341 (2)	0.28606 (2)	0.01520 (6)	
S1	0.15381 (2)	0.14511 (4)	0.35017 (2)	0.02303 (12)	
S2	0.18175 (2)	0.15280 (4)	0.20811 (2)	0.01718 (10)	
N1	0.11691 (5)	0.24993 (13)	0.41537 (7)	0.0209 (4)	
H1N	0.1105 (6)	0.3059 (10)	0.4281 (8)	0.025*	
N2	0.14118 (4)	0.34387 (12)	0.35625 (6)	0.0167 (3)	
N3	0.16200 (4)	0.35198 (12)	0.31407 (6)	0.0151 (3)	
N4	0.23674 (4)	0.17761 (14)	0.15053 (6)	0.0215 (4)	
H4N	0.2574 (3)	0.2004 (17)	0.1460 (9)	0.026*	
N5	0.24781 (4)	0.22317 (12)	0.23164 (6)	0.0153 (3)	
N6	0.23457 (4)	0.24038 (12)	0.27890 (6)	0.0138 (3)	
C1	0.13684 (5)	0.25411 (15)	0.37405 (7)	0.0168 (4)	
C2	0.10863 (6)	0.16246 (16)	0.44418 (8)	0.0240 (5)	
H2A	0.1050	0.1069	0.4205	0.029*	
H2B	0.0866	0.1732	0.4624	0.029*	
C3	0.13712 (6)	0.1363 (2)	0.48203 (10)	0.0404 (6)	
H3A	0.1590	0.1260	0.4642	0.061*	
H3B	0.1308	0.0761	0.4999	0.061*	
H3C	0.1401	0.1898	0.5065	0.061*	
C4	0.17428 (5)	0.44016 (14)	0.30504 (7)	0.0151 (4)	
C5	0.17094 (5)	0.52141 (15)	0.34000 (7)	0.0163 (4)	
H5	0.1634	0.5076	0.3734	0.020*	
C6	0.17784 (5)	0.61502 (15)	0.32811 (7)	0.0170 (4)	
H6	0.1847	0.6271	0.2942	0.020*	
C7	0.17592 (5)	0.70083 (15)	0.36164 (8)	0.0169 (4)	
C8	0.18236 (5)	0.79338 (15)	0.34113 (8)	0.0194 (4)	
H8	0.1871	0.7992	0.3060	0.023*	
C9	0.18193 (5)	0.87665 (16)	0.37105 (9)	0.0239 (5)	
H9	0.1861	0.9391	0.3564	0.029*	
C10	0.17540 (6)	0.86904 (16)	0.42231 (9)	0.0263 (5)	
H10	0.1753	0.9260	0.4430	0.032*	
C11	0.16894 (6)	0.77787 (17)	0.44327 (8)	0.0274 (5)	
H11	0.1644	0.7727	0.4785	0.033*	
C12	0.16906 (5)	0.69427 (16)	0.41363 (8)	0.0221 (5)	
H12	0.1645	0.6322	0.4285	0.026*	
C13	0.19356 (5)	0.45041 (14)	0.25683 (7)	0.0141 (4)	
C14	0.17819 (5)	0.41984 (15)	0.21118 (7)	0.0177 (4)	
H14	0.1549	0.3964	0.2106	0.021*	
C15	0.19675 (6)	0.42343 (15)	0.16651 (8)	0.0210 (4)	
H15	0.1861	0.4036	0.1354	0.025*	
C16	0.23107 (5)	0.45622 (15)	0.16752 (8)	0.0205 (4)	
H16	0.2441	0.4569	0.1372	0.025*	
C17	0.24639 (5)	0.48789 (15)	0.21253 (8)	0.0188 (4)	
H17	0.2698	0.5109	0.2129	0.023*	
C18	0.22768 (5)	0.48614 (14)	0.25713 (7)	0.0156 (4)	
H18	0.2381	0.5092	0.2879	0.019*	
C19	0.22494 (5)	0.18887 (14)	0.19800 (7)	0.0158 (4)	
C20	0.21685 (6)	0.1434 (2)	0.10634 (8)	0.0307 (5)	
H20A	0.1958	0.1847	0.1016	0.037*	
H20B	0.2091	0.0751	0.1121	0.037*	
C21	0.23816 (8)	0.1477 (3)	0.05985 (9)	0.0517 (8)	
H21A	0.2450	0.2157	0.0534	0.078*	
H21B	0.2245	0.1227	0.0309	0.078*	
H21C	0.2591	0.1073	0.0647	0.078*	
C22	0.25651 (5)	0.27184 (14)	0.31400 (7)	0.0159 (4)	
C23	0.24220 (5)	0.29792 (15)	0.36244 (7)	0.0188 (4)	
H23	0.2179	0.2878	0.3665	0.023*	
C24	0.25988 (6)	0.33495 (17)	0.40216 (8)	0.0239 (5)	
H24	0.2843	0.3432	0.3990	0.029*	
C25	0.24426 (6)	0.36381 (17)	0.45037 (8)	0.0261 (5)	
C26	0.20847 (6)	0.37796 (18)	0.45568 (8)	0.0300 (5)	
H26	0.1933	0.3694	0.4271	0.036*	
C27	0.19487 (7)	0.4042 (2)	0.50186 (9)	0.0386 (6)	
H27	0.1705	0.4139	0.5048	0.046*	
C28	0.21669 (7)	0.4165 (2)	0.54402 (9)	0.0422 (7)	
H28	0.2073	0.4339	0.5759	0.051*	
C29	0.25221 (7)	0.4033 (2)	0.53937 (9)	0.0420 (7)	
H29	0.2672	0.4112	0.5681	0.050*	
C30	0.26593 (7)	0.3784 (2)	0.49281 (9)	0.0354 (6)	
H30	0.2904	0.3713	0.4897	0.043*	
C31	0.29471 (5)	0.28225 (15)	0.30474 (7)	0.0174 (4)	
C32	0.31570 (5)	0.19919 (16)	0.30292 (7)	0.0201 (4)	
H32	0.3059	0.1363	0.3086	0.024*	
C33	0.35100 (6)	0.20793 (17)	0.29278 (8)	0.0251 (5)	
H33	0.3652	0.1510	0.2919	0.030*	
C34	0.36554 (6)	0.29898 (18)	0.28396 (9)	0.0297 (5)	
H34	0.3895	0.3045	0.2759	0.036*	
C35	0.34490 (6)	0.38236 (18)	0.28701 (9)	0.0281 (5)	
H35	0.3549	0.4452	0.2818	0.034*	
C36	0.30974 (5)	0.37415 (16)	0.29771 (8)	0.0218 (4)	
H36	0.2958	0.4315	0.3003	0.026*	
Zn2	0.09256 (2)	0.62509 (2)	0.50259 (2)	0.01479 (6)	
S3	0.09646 (2)	0.48949 (4)	0.45303 (2)	0.01870 (11)	
S4	0.13339 (2)	0.67287 (4)	0.56165 (2)	0.01954 (11)	
N7	0.08819 (4)	0.49976 (13)	0.35350 (6)	0.0174 (4)	
H7N	0.1005 (5)	0.4464 (11)	0.3560 (8)	0.021*	
N8	0.07995 (4)	0.64722 (12)	0.39142 (6)	0.0158 (3)	
N9	0.08139 (4)	0.70030 (12)	0.43591 (6)	0.0154 (3)	
N10	0.11345 (5)	0.74709 (17)	0.64894 (7)	0.0315 (5)	
H10N	0.1359 (3)	0.7553 (19)	0.6528 (9)	0.038*	
N11	0.06752 (4)	0.70684 (13)	0.59836 (6)	0.0185 (4)	
N12	0.05551 (4)	0.66810 (12)	0.55301 (6)	0.0150 (3)	
C37	0.08771 (5)	0.55407 (15)	0.39636 (7)	0.0151 (4)	
C38	0.08438 (6)	0.54449 (17)	0.30322 (7)	0.0220 (5)	
H38A	0.0963	0.5030	0.2781	0.026*	
H38B	0.0959	0.6095	0.3035	0.026*	
C39	0.04633 (6)	0.5567 (2)	0.28717 (8)	0.0317 (5)	
H39A	0.0351	0.4922	0.2855	0.048*	
H39B	0.0449	0.5878	0.2537	0.048*	
H39C	0.0344	0.5978	0.3119	0.048*	
C40	0.07469 (5)	0.79387 (15)	0.43138 (7)	0.0157 (4)	
C41	0.07700 (5)	0.85264 (15)	0.47690 (7)	0.0181 (4)	
H41	0.0890	0.8251	0.5053	0.022*	
C42	0.06363 (5)	0.94259 (15)	0.48250 (7)	0.0191 (4)	
H42	0.0524	0.9716	0.4538	0.023*	
C43	0.06507 (6)	0.99939 (16)	0.52936 (8)	0.0234 (5)	
C44	0.04271 (7)	1.07947 (17)	0.53472 (9)	0.0335 (6)	
H44	0.0270	1.0961	0.5078	0.040*	
C45	0.04319 (10)	1.1348 (2)	0.57862 (11)	0.0568 (9)	
H45	0.0276	1.1884	0.5819	0.068*	
C46	0.06614 (12)	1.1125 (2)	0.61746 (11)	0.0708 (12)	
H46	0.0666	1.1511	0.6474	0.085*	
C47	0.08860 (10)	1.0336 (2)	0.61298 (10)	0.0592 (10)	
H47	0.1044	1.0182	0.6400	0.071*	
C48	0.08810 (7)	0.97724 (18)	0.56942 (8)	0.0348 (6)	
H48	0.1035	0.9231	0.5667	0.042*	
C49	0.06507 (5)	0.83867 (15)	0.38153 (7)	0.0178 (4)	
C50	0.08664 (6)	0.90851 (16)	0.36002 (8)	0.0221 (5)	
H50	0.1071	0.9298	0.3777	0.027*	
C51	0.07828 (7)	0.94734 (17)	0.31242 (8)	0.0307 (5)	
H51	0.0934	0.9938	0.2973	0.037*	
C52	0.04817 (8)	0.91847 (19)	0.28721 (9)	0.0383 (6)	
H52	0.0423	0.9456	0.2550	0.046*	
C53	0.02658 (8)	0.8502 (2)	0.30887 (10)	0.0424 (7)	
H53	0.0057	0.8311	0.2917	0.051*	
C54	0.03516 (6)	0.80927 (18)	0.35533 (9)	0.0299 (5)	
H54	0.0205	0.7607	0.3695	0.036*	
C55	0.10145 (5)	0.70964 (16)	0.60429 (7)	0.0189 (4)	
C56	0.09005 (6)	0.7936 (2)	0.68573 (9)	0.0394 (7)	
H56A	0.1035	0.8408	0.7069	0.047*	
H56B	0.0716	0.8304	0.6672	0.047*	
C57	0.07404 (9)	0.7204 (2)	0.71847 (11)	0.0536 (8)	
H57A	0.0610	0.6731	0.6975	0.080*	
H57B	0.0581	0.7530	0.7418	0.080*	
H57C	0.0923	0.6861	0.7380	0.080*	
C58	0.02184 (5)	0.67592 (15)	0.54521 (7)	0.0150 (4)	
C59	0.00695 (5)	0.63366 (15)	0.49947 (7)	0.0163 (4)	
H59	0.0216	0.5953	0.4787	0.020*	
C60	−0.02650 (5)	0.64535 (15)	0.48458 (7)	0.0163 (4)	
H60	−0.0408	0.6829	0.5063	0.020*	
C61	−0.04328 (5)	0.60656 (15)	0.43862 (7)	0.0173 (4)	
C62	−0.02457 (5)	0.56030 (15)	0.40025 (7)	0.0202 (4)	
H62	0.0001	0.5554	0.4031	0.024*	
C63	−0.04178 (6)	0.52170 (17)	0.35816 (8)	0.0253 (5)	
H63	−0.0289	0.4901	0.3324	0.030*	
C64	−0.07776 (6)	0.52884 (18)	0.35333 (8)	0.0283 (5)	
H64	−0.0895	0.5013	0.3246	0.034*	
C65	−0.09651 (6)	0.57606 (18)	0.39027 (8)	0.0266 (5)	
H65	−0.1211	0.5818	0.3868	0.032*	
C66	−0.07939 (5)	0.61493 (16)	0.43230 (8)	0.0221 (5)	
H66	−0.0924	0.6480	0.4574	0.027*	
C67	−0.00047 (5)	0.72828 (15)	0.58235 (7)	0.0152 (4)	
C68	0.00366 (5)	0.82902 (16)	0.58950 (8)	0.0201 (4)	
H68	0.0202	0.8639	0.5701	0.024*	
C69	−0.01618 (6)	0.87864 (16)	0.62458 (8)	0.0227 (5)	
H69	−0.0136	0.9475	0.6288	0.027*	
C70	−0.03976 (6)	0.82737 (16)	0.65346 (8)	0.0236 (5)	
H70	−0.0530	0.8609	0.6781	0.028*	
C71	−0.04412 (5)	0.72761 (16)	0.64660 (8)	0.0222 (5)	
H71	−0.0604	0.6928	0.6665	0.027*	
C72	−0.02476 (5)	0.67807 (15)	0.61072 (8)	0.0192 (4)	
H72	−0.0281	0.6098	0.6056	0.023*	

### Atomic displacement parameters (Å^2^)

**Table:**

	*U*^11^	*U*^22^	*U*^33^	*U*^12^	*U*^13^	*U*^23^
Zn1	0.01408 (11)	0.01313 (12)	0.01848 (11)	0.00079 (9)	0.00292 (9)	−0.00149 (9)
S1	0.0274 (3)	0.0121 (2)	0.0300 (3)	0.0016 (2)	0.0121 (2)	0.0022 (2)
S2	0.0148 (2)	0.0176 (2)	0.0192 (2)	−0.00086 (19)	−0.00143 (19)	−0.0026 (2)
N1	0.0240 (9)	0.0138 (9)	0.0253 (9)	0.0008 (7)	0.0110 (7)	0.0027 (8)
N2	0.0158 (8)	0.0139 (8)	0.0206 (8)	0.0012 (7)	0.0073 (7)	0.0008 (7)
N3	0.0136 (8)	0.0138 (8)	0.0179 (8)	0.0017 (6)	0.0039 (6)	0.0006 (7)
N4	0.0153 (9)	0.0314 (11)	0.0178 (8)	0.0006 (8)	0.0004 (7)	−0.0059 (8)
N5	0.0161 (8)	0.0167 (9)	0.0130 (8)	0.0024 (7)	0.0014 (6)	−0.0020 (7)
N6	0.0145 (8)	0.0132 (8)	0.0137 (8)	0.0014 (6)	0.0011 (6)	−0.0005 (7)
C1	0.0154 (9)	0.0146 (10)	0.0204 (10)	0.0002 (8)	0.0034 (8)	0.0002 (8)
C2	0.0221 (11)	0.0212 (11)	0.0289 (11)	−0.0018 (9)	0.0092 (9)	0.0069 (10)
C3	0.0305 (13)	0.0499 (17)	0.0407 (14)	−0.0037 (12)	0.0011 (11)	0.0204 (13)
C4	0.0116 (9)	0.0147 (10)	0.0189 (10)	0.0018 (7)	0.0014 (7)	0.0015 (8)
C5	0.0145 (9)	0.0181 (10)	0.0165 (9)	−0.0001 (8)	0.0040 (7)	−0.0001 (8)
C6	0.0153 (9)	0.0190 (10)	0.0166 (9)	0.0001 (8)	0.0021 (8)	−0.0019 (8)
C7	0.0126 (9)	0.0155 (10)	0.0228 (10)	0.0000 (8)	0.0040 (8)	−0.0014 (8)
C8	0.0184 (10)	0.0184 (11)	0.0214 (10)	0.0020 (8)	0.0042 (8)	−0.0001 (9)
C9	0.0225 (11)	0.0145 (10)	0.0350 (12)	−0.0024 (9)	0.0058 (9)	0.0005 (10)
C10	0.0243 (11)	0.0201 (11)	0.0347 (12)	−0.0018 (9)	0.0078 (9)	−0.0123 (10)
C11	0.0276 (12)	0.0312 (13)	0.0236 (11)	−0.0059 (10)	0.0070 (9)	−0.0064 (10)
C12	0.0239 (11)	0.0191 (11)	0.0235 (11)	−0.0058 (9)	0.0065 (9)	−0.0008 (9)
C13	0.0163 (9)	0.0087 (9)	0.0174 (9)	0.0012 (7)	0.0014 (7)	0.0013 (8)
C14	0.0169 (10)	0.0138 (10)	0.0225 (10)	−0.0016 (8)	−0.0004 (8)	0.0017 (8)
C15	0.0273 (11)	0.0178 (11)	0.0177 (10)	−0.0005 (9)	−0.0030 (8)	0.0011 (9)
C16	0.0254 (11)	0.0192 (11)	0.0170 (10)	0.0019 (9)	0.0055 (8)	0.0014 (9)
C17	0.0165 (10)	0.0176 (10)	0.0222 (10)	0.0002 (8)	0.0033 (8)	0.0023 (9)
C18	0.0187 (10)	0.0130 (10)	0.0150 (9)	−0.0005 (8)	0.0004 (8)	−0.0004 (8)
C19	0.0152 (9)	0.0133 (10)	0.0188 (10)	0.0034 (8)	−0.0001 (8)	−0.0003 (8)
C20	0.0284 (12)	0.0446 (15)	0.0190 (10)	−0.0011 (11)	−0.0034 (9)	−0.0063 (11)
C21	0.0464 (17)	0.088 (2)	0.0214 (12)	−0.0107 (16)	0.0011 (11)	−0.0106 (14)
C22	0.0181 (10)	0.0125 (10)	0.0171 (9)	0.0022 (8)	−0.0001 (8)	0.0006 (8)
C23	0.0185 (10)	0.0207 (11)	0.0173 (10)	0.0017 (8)	−0.0004 (8)	−0.0010 (9)
C24	0.0213 (11)	0.0280 (12)	0.0224 (11)	0.0044 (9)	−0.0029 (9)	−0.0020 (9)
C25	0.0291 (12)	0.0303 (13)	0.0188 (10)	0.0037 (10)	−0.0021 (9)	−0.0071 (10)
C26	0.0293 (12)	0.0386 (14)	0.0219 (11)	0.0030 (11)	−0.0036 (9)	−0.0076 (10)
C27	0.0323 (14)	0.0544 (18)	0.0293 (13)	0.0014 (12)	0.0050 (10)	−0.0133 (13)
C28	0.0453 (16)	0.0587 (19)	0.0228 (12)	−0.0010 (14)	0.0055 (11)	−0.0167 (13)
C29	0.0453 (16)	0.0574 (18)	0.0230 (12)	0.0018 (14)	−0.0090 (11)	−0.0131 (12)
C30	0.0309 (13)	0.0492 (16)	0.0259 (12)	0.0036 (12)	−0.0050 (10)	−0.0121 (12)
C31	0.0177 (10)	0.0199 (11)	0.0144 (9)	−0.0007 (8)	−0.0047 (8)	−0.0005 (8)
C32	0.0204 (10)	0.0201 (11)	0.0198 (10)	0.0004 (8)	−0.0037 (8)	−0.0011 (9)
C33	0.0200 (11)	0.0282 (12)	0.0269 (11)	0.0085 (9)	−0.0038 (9)	−0.0002 (10)
C34	0.0148 (10)	0.0368 (14)	0.0376 (13)	−0.0014 (10)	−0.0021 (9)	−0.0004 (11)
C35	0.0229 (11)	0.0263 (12)	0.0351 (13)	−0.0058 (10)	−0.0039 (10)	0.0023 (10)
C36	0.0184 (10)	0.0210 (11)	0.0257 (11)	0.0011 (9)	−0.0051 (8)	−0.0027 (9)
Zn2	0.01392 (11)	0.01808 (12)	0.01235 (11)	0.00177 (9)	0.00010 (8)	0.00002 (9)
S3	0.0247 (3)	0.0162 (2)	0.0153 (2)	0.0031 (2)	0.00224 (19)	0.0012 (2)
S4	0.0124 (2)	0.0311 (3)	0.0151 (2)	0.0007 (2)	−0.00026 (18)	0.0001 (2)
N7	0.0209 (9)	0.0161 (9)	0.0155 (8)	0.0032 (7)	0.0034 (7)	−0.0007 (7)
N8	0.0176 (8)	0.0169 (9)	0.0130 (8)	0.0020 (7)	0.0015 (6)	−0.0028 (7)
N9	0.0147 (8)	0.0189 (9)	0.0125 (7)	0.0019 (7)	−0.0004 (6)	−0.0019 (7)
N10	0.0151 (9)	0.0583 (14)	0.0210 (9)	0.0013 (9)	−0.0034 (8)	−0.0149 (10)
N11	0.0154 (8)	0.0270 (10)	0.0132 (8)	0.0012 (7)	−0.0013 (6)	−0.0030 (7)
N12	0.0161 (8)	0.0166 (9)	0.0122 (7)	−0.0002 (7)	−0.0004 (6)	−0.0010 (7)
C37	0.0115 (9)	0.0182 (10)	0.0158 (9)	0.0005 (8)	0.0028 (7)	−0.0009 (8)
C38	0.0265 (11)	0.0252 (12)	0.0144 (9)	0.0023 (9)	0.0029 (8)	−0.0030 (9)
C39	0.0297 (13)	0.0444 (15)	0.0208 (11)	0.0048 (11)	−0.0039 (9)	−0.0037 (11)
C40	0.0132 (9)	0.0185 (10)	0.0156 (9)	0.0018 (8)	0.0010 (7)	−0.0006 (8)
C41	0.0201 (10)	0.0197 (11)	0.0145 (9)	−0.0012 (8)	−0.0022 (8)	0.0020 (8)
C42	0.0213 (10)	0.0203 (11)	0.0155 (9)	−0.0034 (8)	0.0015 (8)	0.0018 (9)
C43	0.0378 (13)	0.0163 (11)	0.0166 (10)	−0.0101 (9)	0.0087 (9)	−0.0013 (9)
C44	0.0567 (16)	0.0177 (11)	0.0269 (12)	−0.0043 (11)	0.0189 (11)	0.0011 (10)
C45	0.116 (3)	0.0187 (13)	0.0368 (16)	−0.0011 (16)	0.0347 (17)	−0.0045 (12)
C46	0.158 (4)	0.0300 (16)	0.0247 (14)	−0.023 (2)	0.0151 (19)	−0.0127 (13)
C47	0.116 (3)	0.0389 (18)	0.0225 (13)	−0.0262 (19)	−0.0109 (15)	−0.0036 (13)
C48	0.0559 (17)	0.0258 (13)	0.0224 (11)	−0.0146 (12)	−0.0042 (11)	−0.0001 (10)
C49	0.0235 (10)	0.0158 (10)	0.0140 (9)	0.0056 (8)	−0.0017 (8)	−0.0019 (8)
C50	0.0258 (11)	0.0193 (11)	0.0213 (10)	0.0063 (9)	0.0030 (9)	−0.0003 (9)
C51	0.0472 (15)	0.0202 (12)	0.0250 (11)	0.0106 (11)	0.0119 (11)	0.0057 (10)
C52	0.0688 (19)	0.0274 (13)	0.0181 (11)	0.0112 (13)	−0.0101 (12)	0.0009 (10)
C53	0.0571 (18)	0.0342 (15)	0.0349 (14)	−0.0029 (13)	−0.0286 (13)	0.0050 (12)
C54	0.0344 (13)	0.0257 (12)	0.0292 (12)	−0.0015 (10)	−0.0114 (10)	0.0051 (10)
C55	0.0170 (10)	0.0225 (11)	0.0171 (9)	0.0009 (8)	−0.0008 (8)	0.0000 (9)
C56	0.0274 (13)	0.0653 (19)	0.0252 (12)	−0.0023 (13)	−0.0055 (10)	−0.0137 (13)
C57	0.066 (2)	0.055 (2)	0.0391 (16)	0.0093 (16)	0.0059 (14)	−0.0032 (15)
C58	0.0140 (9)	0.0160 (10)	0.0151 (9)	−0.0012 (8)	0.0009 (7)	0.0016 (8)
C59	0.0173 (10)	0.0168 (10)	0.0149 (9)	−0.0005 (8)	0.0013 (8)	−0.0017 (8)
C60	0.0181 (10)	0.0160 (10)	0.0148 (9)	−0.0003 (8)	0.0017 (8)	−0.0010 (8)
C61	0.0178 (10)	0.0163 (10)	0.0177 (10)	−0.0015 (8)	−0.0020 (8)	0.0032 (8)
C62	0.0195 (10)	0.0215 (11)	0.0194 (10)	−0.0002 (9)	−0.0029 (8)	0.0003 (9)
C63	0.0326 (12)	0.0253 (12)	0.0179 (10)	−0.0010 (10)	−0.0009 (9)	−0.0035 (9)
C64	0.0332 (13)	0.0322 (13)	0.0193 (10)	−0.0073 (10)	−0.0105 (9)	0.0005 (10)
C65	0.0205 (11)	0.0367 (14)	0.0223 (11)	−0.0051 (10)	−0.0070 (9)	0.0061 (10)
C66	0.0202 (10)	0.0274 (12)	0.0187 (10)	0.0000 (9)	0.0001 (8)	0.0038 (9)
C67	0.0127 (9)	0.0197 (10)	0.0131 (9)	0.0017 (8)	−0.0026 (7)	−0.0006 (8)
C68	0.0177 (10)	0.0203 (11)	0.0222 (10)	−0.0014 (8)	0.0012 (8)	0.0005 (9)
C69	0.0278 (11)	0.0148 (10)	0.0254 (11)	0.0024 (9)	−0.0008 (9)	−0.0028 (9)
C70	0.0255 (11)	0.0242 (12)	0.0214 (10)	0.0047 (9)	0.0060 (9)	−0.0045 (9)
C71	0.0212 (11)	0.0227 (11)	0.0228 (10)	−0.0010 (9)	0.0070 (8)	−0.0017 (9)
C72	0.0195 (10)	0.0159 (10)	0.0222 (10)	−0.0004 (8)	0.0004 (8)	−0.0018 (9)

### Geometric parameters (Å, º)

**Table:**

Zn1—N6	2.0522 (16)	Zn2—N12	2.0496 (15)
Zn1—N3	2.0528 (16)	Zn2—N9	2.0727 (16)
Zn1—S2	2.2688 (5)	Zn2—S3	2.2707 (6)
Zn1—S1	2.2827 (6)	Zn2—S4	2.2823 (5)
S1—C1	1.745 (2)	S3—C37	1.761 (2)
S2—C19	1.753 (2)	S4—C55	1.751 (2)
N1—C1	1.344 (2)	N7—C37	1.351 (3)
N1—C2	1.453 (3)	N7—C38	1.464 (3)
N1—H1N	0.871 (10)	N7—H7N	0.870 (9)
N2—C1	1.323 (2)	N8—C37	1.311 (3)
N2—N3	1.385 (2)	N8—N9	1.378 (2)
N3—C4	1.315 (2)	N9—C40	1.307 (3)
N4—C19	1.347 (2)	N10—C55	1.356 (3)
N4—C20	1.457 (3)	N10—C56	1.477 (3)
N4—H4N	0.863 (9)	N10—H10N	0.874 (10)
N5—C19	1.321 (2)	N11—C55	1.309 (2)
N5—N6	1.374 (2)	N11—N12	1.378 (2)
N6—C22	1.311 (2)	N12—C58	1.308 (2)
C2—C3	1.508 (3)	C38—C39	1.521 (3)
C2—H2A	0.9900	C38—H38A	0.9900
C2—H2B	0.9900	C38—H38B	0.9900
C3—H3A	0.9800	C39—H39A	0.9800
C3—H3B	0.9800	C39—H39B	0.9800
C3—H3C	0.9800	C39—H39C	0.9800
C4—C5	1.448 (3)	C40—C41	1.444 (3)
C4—C13	1.489 (3)	C40—C49	1.489 (3)
C5—C6	1.342 (3)	C41—C42	1.339 (3)
C5—H5	0.9500	C41—H41	0.9500
C6—C7	1.469 (3)	C42—C43	1.458 (3)
C6—H6	0.9500	C42—H42	0.9500
C7—C8	1.397 (3)	C43—C48	1.397 (3)
C7—C12	1.403 (3)	C43—C44	1.398 (3)
C8—C9	1.383 (3)	C44—C45	1.381 (4)
C8—H8	0.9500	C44—H44	0.9500
C9—C10	1.383 (3)	C45—C46	1.372 (5)
C9—H9	0.9500	C45—H45	0.9500
C10—C11	1.385 (3)	C46—C47	1.385 (5)
C10—H10	0.9500	C46—H46	0.9500
C11—C12	1.382 (3)	C47—C48	1.382 (4)
C11—H11	0.9500	C47—H47	0.9500
C12—H12	0.9500	C48—H48	0.9500
C13—C14	1.394 (3)	C49—C54	1.387 (3)
C13—C18	1.397 (3)	C49—C50	1.389 (3)
C14—C15	1.388 (3)	C50—C51	1.394 (3)
C14—H14	0.9500	C50—H50	0.9500
C15—C16	1.390 (3)	C51—C52	1.379 (4)
C15—H15	0.9500	C51—H51	0.9500
C16—C17	1.383 (3)	C52—C53	1.377 (4)
C16—H16	0.9500	C52—H52	0.9500
C17—C18	1.389 (3)	C53—C54	1.380 (3)
C17—H17	0.9500	C53—H53	0.9500
C18—H18	0.9500	C54—H54	0.9500
C20—C21	1.487 (3)	C56—C57	1.462 (4)
C20—H20A	0.9900	C56—H56A	0.9900
C20—H20B	0.9900	C56—H56B	0.9900
C21—H21A	0.9800	C57—H57A	0.9800
C21—H21B	0.9800	C57—H57B	0.9800
C21—H21C	0.9800	C57—H57C	0.9800
C22—C23	1.443 (3)	C58—C59	1.444 (3)
C22—C31	1.497 (3)	C58—C67	1.494 (3)
C23—C24	1.336 (3)	C59—C60	1.344 (3)
C23—H23	0.9500	C59—H59	0.9500
C24—C25	1.469 (3)	C60—C61	1.460 (3)
C24—H24	0.9500	C60—H60	0.9500
C25—C26	1.396 (3)	C61—C66	1.397 (3)
C25—C30	1.395 (3)	C61—C62	1.401 (3)
C26—C27	1.380 (3)	C62—C63	1.384 (3)
C26—H26	0.9500	C62—H62	0.9500
C27—C28	1.389 (3)	C63—C64	1.387 (3)
C27—H27	0.9500	C63—H63	0.9500
C28—C29	1.382 (4)	C64—C65	1.380 (3)
C28—H28	0.9500	C64—H64	0.9500
C29—C30	1.386 (3)	C65—C66	1.383 (3)
C29—H29	0.9500	C65—H65	0.9500
C30—H30	0.9500	C66—H66	0.9500
C31—C32	1.391 (3)	C67—C72	1.386 (3)
C31—C36	1.393 (3)	C67—C68	1.395 (3)
C32—C33	1.391 (3)	C68—C69	1.385 (3)
C32—H32	0.9500	C68—H68	0.9500
C33—C34	1.383 (3)	C69—C70	1.383 (3)
C33—H33	0.9500	C69—H69	0.9500
C34—C35	1.389 (3)	C70—C71	1.382 (3)
C34—H34	0.9500	C70—H70	0.9500
C35—C36	1.388 (3)	C71—C72	1.389 (3)
C35—H35	0.9500	C71—H71	0.9500
C36—H36	0.9500	C72—H72	0.9500
			
S1—Zn1—S2	118.67 (2)	S3—Zn2—S4	124.97 (2)
S1—Zn1—N3	87.25 (5)	S3—Zn2—N9	85.99 (5)
S1—Zn1—N6	126.78 (5)	S3—Zn2—N12	131.29 (5)
S2—Zn1—N3	134.00 (5)	S4—Zn2—N9	124.42 (5)
S2—Zn1—N6	87.00 (5)	S4—Zn2—N12	87.23 (5)
N3—Zn1—N6	107.95 (6)	N9—Zn2—N12	105.85 (6)
C1—S1—Zn1	92.96 (7)	C37—S3—Zn2	93.84 (7)
C19—S2—Zn1	91.99 (7)	C55—S4—Zn2	92.29 (7)
C1—N1—C2	126.29 (18)	C37—N7—C38	121.72 (18)
C1—N1—H1N	116.4 (15)	C37—N7—H7N	114.2 (15)
C2—N1—H1N	116.8 (15)	C38—N7—H7N	117.6 (15)
C1—N2—N3	115.89 (16)	C37—N8—N9	114.79 (16)
C4—N3—N2	115.61 (16)	C40—N9—N8	115.50 (16)
C4—N3—Zn1	125.47 (12)	C40—N9—Zn2	126.72 (13)
N2—N3—Zn1	116.26 (12)	N8—N9—Zn2	117.76 (12)
C19—N4—C20	126.77 (18)	C55—N10—C56	122.08 (18)
C19—N4—H4N	114.5 (16)	C55—N10—H10N	117.9 (17)
C20—N4—H4N	118.2 (16)	C56—N10—H10N	118.7 (17)
C19—N5—N6	114.62 (16)	C55—N11—N12	115.50 (16)
C22—N6—N5	116.88 (16)	C58—N12—N11	114.72 (15)
C22—N6—Zn1	126.45 (13)	C58—N12—Zn2	128.03 (13)
N5—N6—Zn1	116.52 (12)	N11—N12—Zn2	116.56 (12)
N2—C1—N1	113.83 (17)	N8—C37—N7	117.06 (18)
N2—C1—S1	127.62 (15)	N8—C37—S3	127.47 (15)
N1—C1—S1	118.55 (15)	N7—C37—S3	115.44 (15)
N1—C2—C3	112.14 (19)	N7—C38—C39	112.11 (17)
N1—C2—H2A	109.2	N7—C38—H38A	109.2
C3—C2—H2A	109.2	C39—C38—H38A	109.2
N1—C2—H2B	109.2	N7—C38—H38B	109.2
C3—C2—H2B	109.2	C39—C38—H38B	109.2
H2A—C2—H2B	107.9	H38A—C38—H38B	107.9
C2—C3—H3A	109.5	C38—C39—H39A	109.5
C2—C3—H3B	109.5	C38—C39—H39B	109.5
H3A—C3—H3B	109.5	H39A—C39—H39B	109.5
C2—C3—H3C	109.5	C38—C39—H39C	109.5
H3A—C3—H3C	109.5	H39A—C39—H39C	109.5
H3B—C3—H3C	109.5	H39B—C39—H39C	109.5
N3—C4—C5	123.26 (17)	N9—C40—C41	117.21 (18)
N3—C4—C13	115.20 (17)	N9—C40—C49	121.76 (18)
C5—C4—C13	121.49 (17)	C41—C40—C49	121.04 (18)
C6—C5—C4	124.01 (18)	C42—C41—C40	125.62 (19)
C6—C5—H5	118.0	C42—C41—H41	117.2
C4—C5—H5	118.0	C40—C41—H41	117.2
C5—C6—C7	127.24 (18)	C41—C42—C43	124.9 (2)
C5—C6—H6	116.4	C41—C42—H42	117.6
C7—C6—H6	116.4	C43—C42—H42	117.6
C8—C7—C12	118.24 (18)	C48—C43—C44	118.2 (2)
C8—C7—C6	118.42 (17)	C48—C43—C42	122.6 (2)
C12—C7—C6	123.30 (18)	C44—C43—C42	119.2 (2)
C9—C8—C7	121.16 (19)	C45—C44—C43	120.8 (3)
C9—C8—H8	119.4	C45—C44—H44	119.6
C7—C8—H8	119.4	C43—C44—H44	119.6
C10—C9—C8	120.0 (2)	C46—C45—C44	120.2 (3)
C10—C9—H9	120.0	C46—C45—H45	119.9
C8—C9—H9	120.0	C44—C45—H45	119.9
C9—C10—C11	119.6 (2)	C45—C46—C47	120.0 (3)
C9—C10—H10	120.2	C45—C46—H46	120.0
C11—C10—H10	120.2	C47—C46—H46	120.0
C12—C11—C10	120.9 (2)	C48—C47—C46	120.3 (3)
C12—C11—H11	119.6	C48—C47—H47	119.9
C10—C11—H11	119.6	C46—C47—H47	119.9
C11—C12—C7	120.1 (2)	C47—C48—C43	120.4 (3)
C11—C12—H12	119.9	C47—C48—H48	119.8
C7—C12—H12	119.9	C43—C48—H48	119.8
C14—C13—C18	119.38 (17)	C54—C49—C50	119.25 (19)
C14—C13—C4	120.00 (17)	C54—C49—C40	120.62 (19)
C18—C13—C4	120.55 (17)	C50—C49—C40	120.10 (18)
C15—C14—C13	120.42 (19)	C49—C50—C51	119.9 (2)
C15—C14—H14	119.8	C49—C50—H50	120.0
C13—C14—H14	119.8	C51—C50—H50	120.0
C14—C15—C16	119.71 (19)	C52—C51—C50	120.1 (2)
C14—C15—H15	120.1	C52—C51—H51	119.9
C16—C15—H15	120.1	C50—C51—H51	119.9
C17—C16—C15	120.26 (18)	C53—C52—C51	119.9 (2)
C17—C16—H16	119.9	C53—C52—H52	120.1
C15—C16—H16	119.9	C51—C52—H52	120.1
C16—C17—C18	120.16 (19)	C52—C53—C54	120.4 (2)
C16—C17—H17	119.9	C52—C53—H53	119.8
C18—C17—H17	119.9	C54—C53—H53	119.8
C17—C18—C13	120.02 (18)	C53—C54—C49	120.4 (2)
C17—C18—H18	120.0	C53—C54—H54	119.8
C13—C18—H18	120.0	C49—C54—H54	119.8
N5—C19—N4	115.66 (17)	N11—C55—N10	115.82 (18)
N5—C19—S2	127.95 (15)	N11—C55—S4	128.40 (15)
N4—C19—S2	116.36 (15)	N10—C55—S4	115.77 (15)
N4—C20—C21	111.0 (2)	C57—C56—N10	111.2 (2)
N4—C20—H20A	109.4	C57—C56—H56A	109.4
C21—C20—H20A	109.4	N10—C56—H56A	109.4
N4—C20—H20B	109.4	C57—C56—H56B	109.4
C21—C20—H20B	109.4	N10—C56—H56B	109.4
H20A—C20—H20B	108.0	H56A—C56—H56B	108.0
C20—C21—H21A	109.5	C56—C57—H57A	109.5
C20—C21—H21B	109.5	C56—C57—H57B	109.5
H21A—C21—H21B	109.5	H57A—C57—H57B	109.5
C20—C21—H21C	109.5	C56—C57—H57C	109.5
H21A—C21—H21C	109.5	H57A—C57—H57C	109.5
H21B—C21—H21C	109.5	H57B—C57—H57C	109.5
N6—C22—C23	117.06 (17)	N12—C58—C59	118.20 (17)
N6—C22—C31	122.24 (17)	N12—C58—C67	120.79 (17)
C23—C22—C31	120.69 (17)	C59—C58—C67	121.00 (17)
C24—C23—C22	126.2 (2)	C60—C59—C58	123.84 (18)
C24—C23—H23	116.9	C60—C59—H59	118.1
C22—C23—H23	116.9	C58—C59—H59	118.1
C23—C24—C25	124.7 (2)	C59—C60—C61	127.06 (18)
C23—C24—H24	117.6	C59—C60—H60	116.5
C25—C24—H24	117.6	C61—C60—H60	116.5
C26—C25—C30	118.2 (2)	C66—C61—C62	118.10 (18)
C26—C25—C24	122.8 (2)	C66—C61—C60	119.34 (18)
C30—C25—C24	119.0 (2)	C62—C61—C60	122.56 (18)
C27—C26—C25	120.9 (2)	C63—C62—C61	120.5 (2)
C27—C26—H26	119.6	C63—C62—H62	119.8
C25—C26—H26	119.6	C61—C62—H62	119.8
C26—C27—C28	120.3 (2)	C62—C63—C64	120.4 (2)
C26—C27—H27	119.9	C62—C63—H63	119.8
C28—C27—H27	119.9	C64—C63—H63	119.8
C29—C28—C27	119.6 (2)	C65—C64—C63	119.9 (2)
C29—C28—H28	120.2	C65—C64—H64	120.0
C27—C28—H28	120.2	C63—C64—H64	120.0
C28—C29—C30	120.1 (2)	C64—C65—C66	119.9 (2)
C28—C29—H29	119.9	C64—C65—H65	120.1
C30—C29—H29	119.9	C66—C65—H65	120.1
C29—C30—C25	120.9 (2)	C65—C66—C61	121.3 (2)
C29—C30—H30	119.5	C65—C66—H66	119.4
C25—C30—H30	119.5	C61—C66—H66	119.4
C32—C31—C36	119.14 (19)	C72—C67—C68	119.29 (18)
C32—C31—C22	119.82 (18)	C72—C67—C58	121.08 (18)
C36—C31—C22	121.04 (18)	C68—C67—C58	119.61 (18)
C33—C32—C31	120.2 (2)	C69—C68—C67	120.59 (19)
C33—C32—H32	119.9	C69—C68—H68	119.7
C31—C32—H32	119.9	C67—C68—H68	119.7
C34—C33—C32	120.4 (2)	C70—C69—C68	119.6 (2)
C34—C33—H33	119.8	C70—C69—H69	120.2
C32—C33—H33	119.8	C68—C69—H69	120.2
C33—C34—C35	119.6 (2)	C71—C70—C69	120.3 (2)
C33—C34—H34	120.2	C71—C70—H70	119.8
C35—C34—H34	120.2	C69—C70—H70	119.8
C36—C35—C34	120.2 (2)	C70—C71—C72	120.2 (2)
C36—C35—H35	119.9	C70—C71—H71	119.9
C34—C35—H35	119.9	C72—C71—H71	119.9
C35—C36—C31	120.4 (2)	C67—C72—C71	120.02 (19)
C35—C36—H36	119.8	C67—C72—H72	120.0
C31—C36—H36	119.8	C71—C72—H72	120.0
			
C1—N2—N3—C4	−161.61 (18)	C37—N8—N9—C40	178.13 (17)
C1—N2—N3—Zn1	0.9 (2)	C37—N8—N9—Zn2	−3.3 (2)
C19—N5—N6—C22	178.11 (17)	C55—N11—N12—C58	−172.63 (18)
C19—N5—N6—Zn1	−6.0 (2)	C55—N11—N12—Zn2	−1.4 (2)
N3—N2—C1—N1	179.06 (17)	N9—N8—C37—N7	−177.12 (16)
N3—N2—C1—S1	−0.8 (3)	N9—N8—C37—S3	4.9 (3)
C2—N1—C1—N2	−177.69 (19)	C38—N7—C37—N8	7.1 (3)
C2—N1—C1—S1	2.2 (3)	C38—N7—C37—S3	−174.65 (14)
Zn1—S1—C1—N2	0.30 (19)	Zn2—S3—C37—N8	−3.59 (18)
Zn1—S1—C1—N1	−179.60 (16)	Zn2—S3—C37—N7	178.41 (14)
C1—N1—C2—C3	82.8 (3)	C37—N7—C38—C39	−86.1 (2)
N2—N3—C4—C5	8.6 (3)	N8—N9—C40—C41	−178.13 (16)
Zn1—N3—C4—C5	−152.08 (15)	Zn2—N9—C40—C41	3.5 (3)
N2—N3—C4—C13	−173.99 (16)	N8—N9—C40—C49	1.8 (3)
Zn1—N3—C4—C13	25.3 (2)	Zn2—N9—C40—C49	−176.55 (14)
N3—C4—C5—C6	−168.14 (19)	N9—C40—C41—C42	−163.6 (2)
C13—C4—C5—C6	14.6 (3)	C49—C40—C41—C42	16.4 (3)
C4—C5—C6—C7	−178.40 (18)	C40—C41—C42—C43	177.55 (19)
C5—C6—C7—C8	−176.6 (2)	C41—C42—C43—C48	17.1 (3)
C5—C6—C7—C12	5.6 (3)	C41—C42—C43—C44	−163.1 (2)
C12—C7—C8—C9	−0.2 (3)	C48—C43—C44—C45	−0.5 (3)
C6—C7—C8—C9	−178.13 (19)	C42—C43—C44—C45	179.7 (2)
C7—C8—C9—C10	0.7 (3)	C43—C44—C45—C46	1.0 (4)
C8—C9—C10—C11	−0.7 (3)	C44—C45—C46—C47	−0.8 (5)
C9—C10—C11—C12	0.2 (3)	C45—C46—C47—C48	0.2 (5)
C10—C11—C12—C7	0.3 (3)	C46—C47—C48—C43	0.3 (4)
C8—C7—C12—C11	−0.3 (3)	C44—C43—C48—C47	−0.2 (4)
C6—C7—C12—C11	177.55 (19)	C42—C43—C48—C47	179.7 (2)
N3—C4—C13—C14	52.5 (2)	N9—C40—C49—C54	61.8 (3)
C5—C4—C13—C14	−130.1 (2)	C41—C40—C49—C54	−118.2 (2)
N3—C4—C13—C18	−124.4 (2)	N9—C40—C49—C50	−116.2 (2)
C5—C4—C13—C18	53.0 (3)	C41—C40—C49—C50	63.7 (3)
C18—C13—C14—C15	1.0 (3)	C54—C49—C50—C51	−0.7 (3)
C4—C13—C14—C15	−175.98 (18)	C40—C49—C50—C51	177.40 (19)
C13—C14—C15—C16	1.1 (3)	C49—C50—C51—C52	1.7 (3)
C14—C15—C16—C17	−1.9 (3)	C50—C51—C52—C53	−0.8 (4)
C15—C16—C17—C18	0.7 (3)	C51—C52—C53—C54	−1.0 (4)
C16—C17—C18—C13	1.4 (3)	C52—C53—C54—C49	2.0 (4)
C14—C13—C18—C17	−2.2 (3)	C50—C49—C54—C53	−1.2 (3)
C4—C13—C18—C17	174.75 (18)	C40—C49—C54—C53	−179.2 (2)
N6—N5—C19—N4	175.76 (16)	N12—N11—C55—N10	−179.60 (19)
N6—N5—C19—S2	−6.5 (3)	N12—N11—C55—S4	1.4 (3)
C20—N4—C19—N5	−178.6 (2)	C56—N10—C55—N11	−7.7 (3)
C20—N4—C19—S2	3.4 (3)	C56—N10—C55—S4	171.4 (2)
Zn1—S2—C19—N5	13.04 (19)	Zn2—S4—C55—N11	−0.6 (2)
Zn1—S2—C19—N4	−169.19 (15)	Zn2—S4—C55—N10	−179.63 (17)
C19—N4—C20—C21	175.9 (2)	C55—N10—C56—C57	84.5 (3)
N5—N6—C22—C23	174.90 (16)	N11—N12—C58—C59	−177.58 (17)
Zn1—N6—C22—C23	−0.5 (3)	Zn2—N12—C58—C59	12.4 (3)
N5—N6—C22—C31	−4.2 (3)	N11—N12—C58—C67	3.2 (3)
Zn1—N6—C22—C31	−179.56 (14)	Zn2—N12—C58—C67	−166.85 (14)
N6—C22—C23—C24	−177.1 (2)	N12—C58—C59—C60	−172.94 (19)
C31—C22—C23—C24	1.9 (3)	C67—C58—C59—C60	6.3 (3)
C22—C23—C24—C25	177.8 (2)	C58—C59—C60—C61	178.86 (19)
C23—C24—C25—C26	−16.0 (4)	C59—C60—C61—C66	171.7 (2)
C23—C24—C25—C30	164.3 (2)	C59—C60—C61—C62	−8.0 (3)
C30—C25—C26—C27	−1.0 (4)	C66—C61—C62—C63	−1.9 (3)
C24—C25—C26—C27	179.4 (2)	C60—C61—C62—C63	177.8 (2)
C25—C26—C27—C28	−0.3 (4)	C61—C62—C63—C64	0.3 (3)
C26—C27—C28—C29	0.7 (5)	C62—C63—C64—C65	1.0 (3)
C27—C28—C29—C30	0.4 (5)	C63—C64—C65—C66	−0.9 (3)
C28—C29—C30—C25	−1.7 (4)	C64—C65—C66—C61	−0.7 (3)
C26—C25—C30—C29	2.0 (4)	C62—C61—C66—C65	2.0 (3)
C24—C25—C30—C29	−178.3 (2)	C60—C61—C66—C65	−177.6 (2)
N6—C22—C31—C32	−74.8 (3)	N12—C58—C67—C72	−112.5 (2)
C23—C22—C31—C32	106.1 (2)	C59—C58—C67—C72	68.2 (3)
N6—C22—C31—C36	105.0 (2)	N12—C58—C67—C68	66.0 (3)
C23—C22—C31—C36	−74.1 (3)	C59—C58—C67—C68	−113.2 (2)
C36—C31—C32—C33	−1.8 (3)	C72—C67—C68—C69	0.1 (3)
C22—C31—C32—C33	178.02 (18)	C58—C67—C68—C69	−178.50 (19)
C31—C32—C33—C34	−0.6 (3)	C67—C68—C69—C70	1.3 (3)
C32—C33—C34—C35	2.2 (3)	C68—C69—C70—C71	−1.5 (3)
C33—C34—C35—C36	−1.4 (3)	C69—C70—C71—C72	0.2 (3)
C34—C35—C36—C31	−1.0 (3)	C68—C67—C72—C71	−1.4 (3)
C32—C31—C36—C35	2.6 (3)	C58—C67—C72—C71	177.18 (19)
C22—C31—C36—C35	−177.24 (19)	C70—C71—C72—C67	1.3 (3)

### Hydrogen-bond geometry (Å, º)

*Cg*1 and *Cg*2 are the centroids of the C31–C36 and C13—C18 rings, respectively.

**Table:**

*D*—H···*A*	*D*—H	H···*A*	*D*···*A*	*D*—H···*A*
N1—H1*N*···S3	0.87 (2)	2.65 (2)	3.5077 (19)	170 (2)
N7—H7*N*···N2	0.87 (2)	2.10 (2)	2.941 (2)	164 (2)
N10—H10*N*···S2^i^	0.87 (1)	2.59 (2)	3.318 (2)	142 (2)
C11—H11···S4	0.95	2.86	3.715 (2)	151
C8—H8···*Cg*1^ii^	0.95	2.73	3.608 (2)	154
C32—H32···*Cg*2^iii^	0.95	2.64	3.532 (2)	157

Symmetry codes: (i) −*x*+1/2, *y*+3/2, −*z*+1/2; (ii) *x*, −*y*, *z*−1/2; (iii) *x*, −*y*−1, *z*−1/2.
